# Supplementary material for: Mn3O4 Nanocrystal-Induced Eryptosis Features Ca2+ Overload, ROS and RNS Accumulation, Calpain Activation, Recruitment of Caspases, and Changes in the Lipid Order of Cell Membranes
Source: Int J Mol Sci. 2025 Apr 1;26(7):3284. doi: 10.3390/ijms26073284 (PMC11989249; doi:10.3390/ijms26073284)
Supplement: Supplementary file 1 [file ijms-26-03284-s001.zip › Figure S1.pdf]

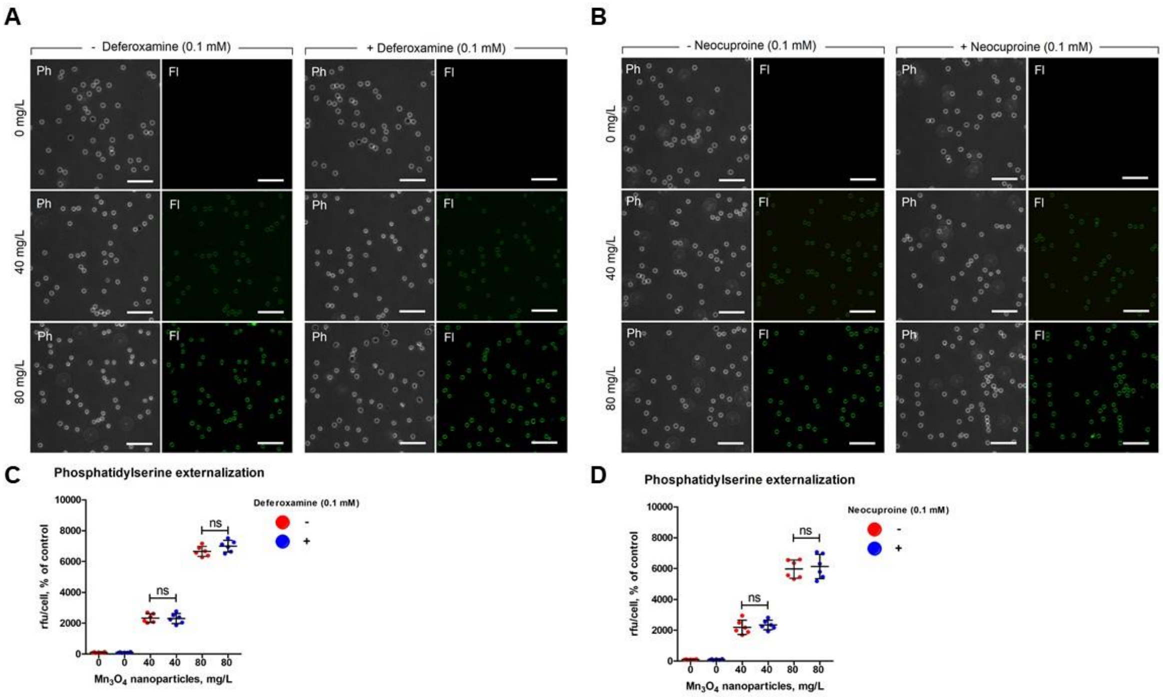

Figure S1. Mn_3O_4 nanoparticles-induced eryptosis is not attenuated by heavy metal (iron and copper) chelators. Laser scanning confocal microscopy-based detection of annexin V-FITC-specific fluorescence to detect phosphatidylserine externalization in erythrocytes treated for 2 h with Mn_3O_4 nanoparticles at 0 mg/L (control samples), 40 mg/L, 80 mg/L and with/without iron-chelating deferoxamine (panel A, C) and copper-chelating neocuproine (panel B, D) at 0.1 mM. Scale bar is 50 µm. T test, mean ± SD, n = 6. Note: Ph – phase contrast mode; Fl – a fluorescence mode reflecting the annexin V-FITC-specific fluorescence intensity; ns – non-significant; rfu – relative fluorescence units.
