# Supplementary material for: Mn3O4 Nanocrystal-Induced Eryptosis Features Ca2+ Overload, ROS and RNS Accumulation, Calpain Activation, Recruitment of Caspases, and Changes in the Lipid Order of Cell Membranes
Source: Int J Mol Sci. 2025 Apr 1;26(7):3284. doi: 10.3390/ijms26073284 (PMC11989249; doi:10.3390/ijms26073284)
Supplement: Supplementary file 1 [file ijms-26-03284-s001.zip › Table S1.pdf]

Table S1 Optical emission spectroscopy with inductively coupled plasma (ICP-OES)-based detection of metal impurities in solutions of Mn_3O_4 nanoparticles

Metal	Concentration, mg/L
Al	1.8
As	< 0.2
Ba	0.3
Ca	4.8
Cd	0.005
Co	0.01
Cu	0.2
Fe	0.1
Hg	< 0.1
Mg	1.0
Ni	0.05
Pb	< 0.05
Ti	< 0.2
V	< 0.1
Zn	0.05
